# The Impact of Adjuvant Chemotherapy on Clinical Outcomes in Locally Advanced Rectal Cancer: A CHORD Consortium Analysis

**DOI:** 10.3390/curroncol32070371

**Published:** 2025-06-26

**Authors:** Kaveh Farrokhi, Horia Marginean, Anas Al Ghamdi, Essa Al Mansor, Shaan Dudani, Rachel A. Goodwin, Timothy R. Asmis, Erin Powell, Patricia A. Tang, Richard Lee-Ying, Michael M. Vickers

**Affiliations:** 1 The Ottawa Hospital, University of Ottawa, 501 Smyth Road, Ottawa, ON K1H 8L6, Canadadr.essa1989@hotmail.com (E.A.M.);; 2 Ottawa Hospital Research Institute, Ottawa, ON K1Y 4E9, Canada; hmarginean@ohri.ca; 3 William Osler Health System, Brampton, ON L6R 3J7, Canada; 4 Dr. H. Bliss Murphy Cancer Centre, St. John’s, Newfoundland and Labrador, NL A1B 3X5, Canada; 5 Arthur J.E. Child Comprehensive Cancer Centre, University of Calgary, Calgary, AB T2N 4N1, Canada; patricia.tang@albertahealthservices.ca (P.A.T.);

**Keywords:** rectal cancer, adjuvant, chemotherapy, real world, outcomes

## Abstract

Background: The impact of adjuvant chemotherapy (AC) on outcomes in real-world patients with locally advanced rectal cancer (LARC) remains uncertain. Methods: Consecutive patients with LARC (stage II/III) undergoing neoadjuvant chemoradiation before curative-intent surgery from 2005 to 2013 were identified in the Canadian Health Outcomes Research Database. The impact of AC on clinical outcomes, including disease-free survival (DFS) and overall survival (OS), was evaluated using the Kaplan–Meier method and Cox proportional hazards modeling. Results: A total of 1448 patients had sufficient data available to be included for analysis with 1085 (74.9%) receiving AC. Of AC patients, 40.5% received oxaliplatin-based treatments. With a median follow-up of 66.43 months, the 5-year DFS rate was 67.7% (95% CI: 64.5–70.1%) vs. 58.7% (95% CI: 52.8–64.2%) in the AC group and non-AC group, respectively (*p* < 0.001). The 5-year OS rate of the whole cohort was 74.3% (95% CI: 71.5–76.85%) while the 5-year OS rate of the AC group was 77.8% (95% CI: 74.7–80.6%) compared with 63.8% (95% CI: 57.9–69.2%) for the non-AC group (*p* < 0.001). On multivariate analysis, patients who received AC had improved DFS (HR 0.6, 95% CI: 0.49–0.73, *p* < 0.001) and OS (HR 0.46, 95% CI: 0.36–0.58, *p* < 0.001). Conclusions: This large multi-institutional database analysis supports the use of AC in real-world LARC patients treated with nCRT followed by surgical resection.

## Simple Summary

Adjuvant chemotherapy remains an open question in the treatment of locally advanced rectal cancer, with conflicting data on the benefit to patient outcomes. In this study we use a large Canadian database to retrospectively examine patients with locally advanced rectal cancer who received adjuvant chemotherapy after initial neoadjuvant chemoradiotherapy and surgical management. We determine that there was a benefit to overall survival and disease-free survival in the patients who received adjuvant chemotherapy compared to patients who did not. Additionally, to identify patients who may benefit from adjuvant chemotherapy, we used multivariate analysis to determine variables associated with improved outcomes. While we did identify variables suggestive of worse prognosis, we did not identify specific variables associated with benefit. This work provides the basis for future randomized trials to determine ideal chemotherapy regimens and further identify patient-specific characteristics predictive of benefit for the use of adjuvant chemotherapy in locally advanced rectal cancer.

## 1. Introduction

Globally, colorectal cancer accounts for nearly 1 in 10 of both new cancer cases and cancer-related deaths [1]. In Canada, colorectal cancer (CRC) is estimated to be the fourth most incident cancer and ranks second for cancer deaths in 2024 [2]. Although the incidence and mortality rates have declined in North America since early 2000, there is an increasing trend towards early-onset CRC with approximately 10% developing before age 50. Similar trends have been seen in high-income nations with increasing prevalence in this group [3,4].

Rectal cancer is a distinct clinical entity within CRC, and in Canada there exists interprovincial heterogeneity in management practices [5,6,7]. Rectal cancer accounts for approximately 29% of all CRC cases and the majority are diagnosed as locally advanced disease [8]. The treatment of locally advanced rectal cancer (LARC) has evolved over the past two decades. A major advancement involved the use of preoperative chemoradiation (nCRT) which resulted in improved local recurrence rates and lower complication rates compared with postoperative CRT in the era of total mesorectal excision (TME) [9]. Subsequent work has expanded on this to show favorable outcomes with preoperative short-course radiotherapy (SCRT) protocols [10,11,12]. Due to the benefits of neoadjuvant treatments, more recent investigations have expanded interest in total neoadjuvant therapy (TNT) compared with nCRT in the treatment of LARC. This TNT approach incorporates systemic chemotherapy and chemoradiation prior to surgery. Several of these trials have demonstrated improvements in distant metastases, disease-free survival (DFS), and/or overall survival (OS) but at a possible risk of higher toxicity and overtreatment [13,14,15]. As a consequence, nCRT followed by TME surgery remains an important treatment strategy and results in low rates of local recurrence (5–10%), with distant recurrence remaining as the primary cause of mortality (~25%) [16,17,18]. To address this distant recurrence risk, adjuvant (postoperative) chemotherapy (AC) has been explored in LARC [19,20]. In contrast to the established role of AC in colon cancer, the evidence for its role in LARC is controversial. In fact, most AC trials have not been able to demonstrate a significant benefit on DFS or OS [21,22,23,24]. Further, subsequent meta-analysis of clinical trials of AC for patients who received preoperative chemoradiation and surgery failed to show improvements in DFS and OS [25]. Of note, these trials suffered from poor AC completion rates, ranging from 42.9–73.6% with the majority near 50% or below [25,26].

An exception to these findings was the ADORE trial that randomized patients in the postoperative setting after completion of neoadjuvant CRT and recovery from TME surgery. This trial showed a 6-year DFS benefit of adjuvant FOLFOX over fluorouracil in patients with ypStage II/III by intention-to-treat (ITT) analysis. Subgroup analysis suggested greater benefit for ypStage III (especially ypN2), high-grade histology, minimally regressed tumor, and those with the absence of lympho-vascular or perineural invasion [27]. Notably, OS was not significantly improved in the ITT population although patients with ypN2 and minimally regressed tumors may have experienced more benefit with FOLFOX.

As a result of this conflicting evidence, guidelines differ with respect to the role of adjuvant chemotherapy in individuals with LARC [28,29,30]. Due to this uncertain benefit in clinical trials, we performed a large, real-world, retrospective analysis from the Cancer Health Outcomes Research Database (CHORD) Consortium investigating the role of AC and its impact on clinical outcomes in Canadian patients with LARC who received neoadjuvant CRT and rectal surgery.

## 2. Materials and Methods

### 2.1. Study Design and Patient Selection

Consecutive patients were identified and data were extracted from the CHORD Consortium Rectal Cancer Database, which is a national, multi-institutional registry of consecutive locally advanced rectal cancer patients who have undergone nCRT followed by curative-intent surgery from five academic (British Columbia Cancer Agency, Cross Cancer Institute, Dr. H Bliss Murphy Cancer Centre, The Ottawa Hospital Cancer Centre, Tom Baker Cancer Centre) and four community (Central Alberta Cancer Centre, Grand Prairie Cancer Centre, Jack Ady Cancer Centre, Margery E. Yuill Cancer Centre) cancer centers in Canada. Patients were eligible for inclusion if they had: pathologically confirmed rectal adenocarcinoma; clinical stage II or III disease as per the seventh edition of the American Joint Commission on Cancer (AJCC) staging system [31]; undergone long-course nCRT followed by curative-intent surgery between 2005 and 2013; documented absence of metastases (confirmed by CT or MRI of the abdomen and either chest radiograph or CT of the thorax). Patients were excluded if they had prior treatments for rectal cancer, evidence of metastatic disease, did not receive surgery, or received neoadjuvant radiation alone.

### 2.2. Statistical Analysis

Patient demographics and baseline characteristics are reported using proportions (%) for categorical variables and medians (range) for continuous variables. Outcomes of interest were disease-free survival (DFS), overall survival (OS). Receipt of AC was defined as receiving one or more cycles of postoperative chemotherapy. DFS was defined as time from diagnosis to first event (local recurrence, distant recurrence, or death from any cause) or censored at the date of last follow-up. OS was defined as the time from diagnosis to death from any cause or censored at the date of last follow-up. Pathological complete response (pCR) was defined as the absence of any residual tumor cells on postoperative histologic evaluation of the rectal surgical specimen. Downstaging was defined as improvement in pathologic TNM staging compared with pretreatment clinical TNM staging. DFS and OS were evaluated using the Kaplan–Meier method. Univariate and multivariate logistic regression and Cox proportional hazard models were used to assess for an association between baseline variables (selected a priori) and outcomes of interest. Factors that were significant at the 0.2 level were retained for analysis in the multivariate model. Estimates (hazards ratios, odds ratios) are presented with 95% confidence intervals (95% CIs). We considered a *p*-value of <0.05 to be significant. All statistical analyses were performed using Stata^®^ software, version 13.1 (Stata Corp LP, College Station, TX, USA).

## 3. Results

### 3.1. Patient and Tumor Characteristics

Of 1527 identified patients with stage II or III rectal cancer, 1448 had sufficient data available to be included for analysis. In our cohort, 1085 patients (74.9%) received AC while 363 patients (25.1%) did not (Table 1). The most common AC regimens were capecitabine (36.3%), FOLFOX (31.6%), single-agent fluorouracil (22.3%), and CAPOX (7%), with 40.5% of patients receiving oxaliplatin-based AC. The median age of AC patients was 60 years (range 22–86) in comparison to 66 (range 27–92) (*p* < 0.001) in the non-AC group and a total of 34% of AC patients were ≥65 years of age. Patients with lower ECOG PS (*p* = 0.013) were more likely to receive AC as were patients with normal BMI (*p* = 0.001) and those that received a higher dose of neoadjuvant radiation (*p* < 0.001). Further, there were differences in receipt of AC by province. Both AC and non-AC groups had similar baseline characteristics including sex, distance from anal verge, pretreatment CEA levels, clinical stage, type of surgery, Quirke grade, pathological stage, and CRM. It was also noted that patients who achieved less downstaging post-nCRT (*p* = 0.01) and those without a pCR (*p* = 0.007) were more likely to receive AC.

### 3.2. Clinical Outcomes

After a median follow-up time of 66.43 months (65.12–65.5, 95% CI), 8% of patients developed local recurrence, 21.8% developed distant recurrence, while 22% of patients had died. In the AC group, 7.8% developed local pelvic recurrences compared with 8.3% who did not receive AC (*p* = 0.22), and 22.3% in the AC group developed distant recurrences compared with 20.4% in the non-AC group (*p* = 0.44).

**Figure 1:**
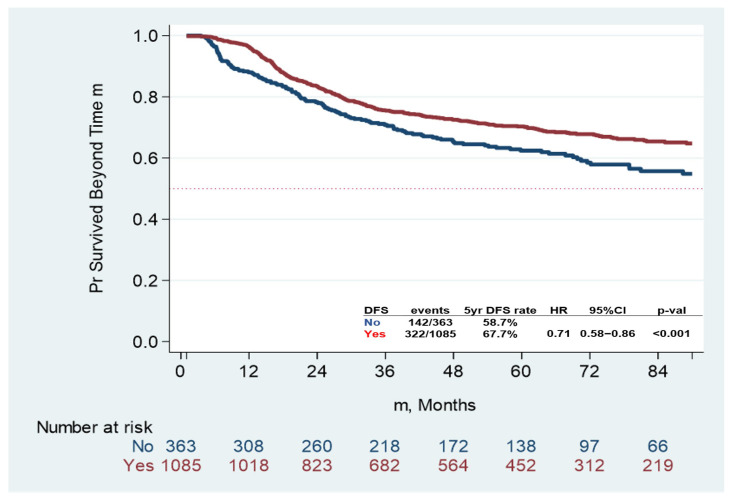
Disease-free survival (DFS) by receipt of adjuvant chemotherapy (Yes/No). Abbreviations: PR, percent; HR, hazard ratio; CI, confidence interval.

The 5-year DFS rate was 65.4% (95% CI: 62.6–68.1%) in all patients. Five-year DFS was 67.7% (95% CI: 64.5–70.1%) and 58.7% (95% CI: 52.8–64.2%) in the AC group and non-AC group, respectively (*p* < 0.001) (Figure 1).

**Figure 2:**
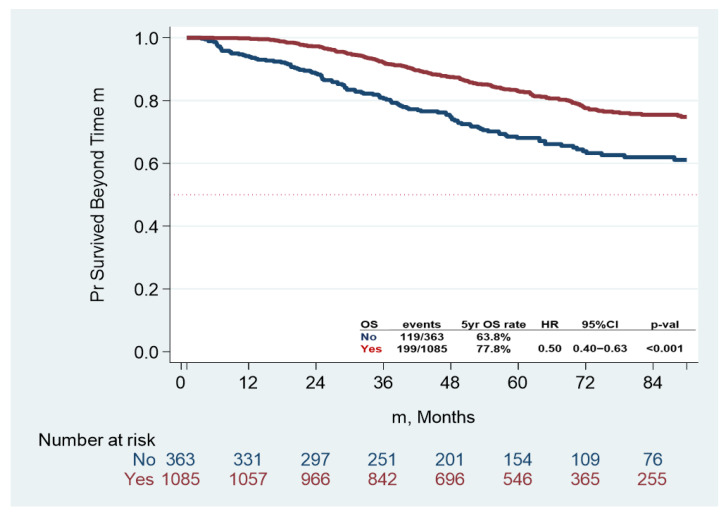
Overall survival (OS) by receipt of adjuvant chemotherapy (Yes/No). Abbreviations: PR, percent; HR, hazard ratio; CI, confidence interval.

The 5-year OS rate of the whole cohort was 74.3% (95% CI: 71.5–76.85%) while the 5-year OS rate of the AC group was 77.8% (95% CI: 74.7–80.6%) compared with 63.8% (95% CI: 57.9–69.2%) for the non-AC group (*p* < 0.001) (Figure 2).

### 3.3. Predictors of DFS

In univariate analysis, AC, province, age at diagnosis, ECOG PS, pretreatment CEA, type of surgery, Quirke grade, CRM involvement, tumor downstaging, and pCR were associated with DFS (Table 2).

**Figure 3:**
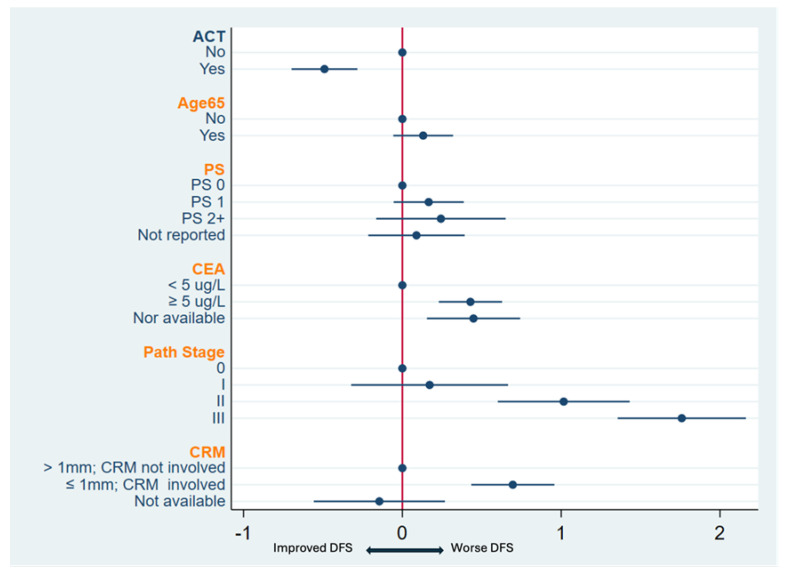
Forest plot of multivariable analysis for disease-free survival (DFS) stratified by province. Abbreviations: ACT, adjuvant chemotherapy; PS, performance scale; CEA, carcinoembryonic antigen; CRM, circumferential resection margin.

In multi-variable analysis, AC (HR 0.6, 95% CI: 0.49–0.73, *p* < 0.001) was associated with improved DFS while higher pretreatment CEA levels (HR 1.53, 95% CI: 1.26–1.87, *p* < 0.001), pathologic stages II (HR 2.75, 95% CI: 1.83–4.13) and III (HR 5.76, 95% CI 3.87–8.57), and involved CRM (HR 2.01, 95% CI: 1.55–2.61) were associated with worse DFS (Figure 3, Table A1). Adjuvant chemotherapy was associated with improved DFS hazard ratios for most categories of pre-CEA levels, pathologic stage, and CRM with significant improvements for those with pretreatment CEA levels ≥5 ug/L, <5 ug/L, those achieving a pCR, and those with a CRM > 1 mm (Table 3).

Oxaliplatin-based chemotherapy was not associated with improved DFS in univariate or multivariable analyses.

### 3.4. Predictors of OS

In univariate analysis, AC, province, age at diagnosis, ECOG PS, distance from anal verge, pretreatment CEA, type of surgery, Quirke grade, pathologic stage, CRM involvement, tumor downstaging, and pCR were associated with OS (Table 2).

**Figure 4:**
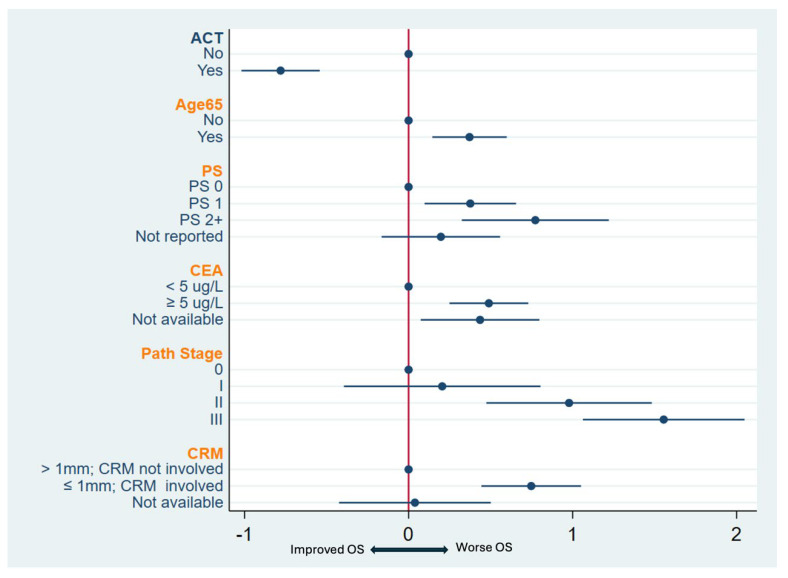
Forest plot of multivariable analysis for overall survival (OS) stratified by province. Abbreviations: ACT, adjuvant chemotherapy; PS, performance scale; CEA, carcinoembryonic antigen; CRM, circumferential resection margin.

In multivariable analysis, AC (HR 0.46, 95% CI: 0.36–0.58, *p* < 0.001) was associated with improved OS while age > 65 years (HR 1.45, 95% CI: 1.16–1.82, *p* = 0.001), ECOG PS I (HR 1.46, 95% CI: 1.10–1.93, *p* = 0.008), ECOG PS II or higher (HR 2.17, 95% CI: 1.38–3.39, *p* = 0.001), higher pretreatment CEA levels (HR 1.63, 95% CI: 1.28–2.08, *p* < 0.001), pathologic stages II (HR 2.66, 95% CI: 1.61–4.41, *p* < 0.001) and III (HR 4.74, 95% CI 2.9–7.76, *p* < 0.001), and involved CRM (HR 2.11, 95% CI: 1.56–2.86, *p* < 0.001) were associated with worse OS (Figure 4, Table A2).

**Figure 5:**
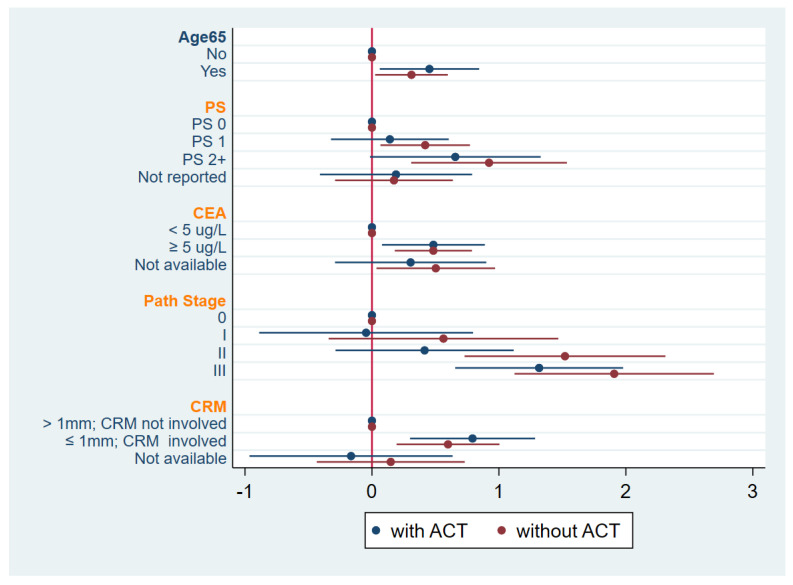
Forest plot of multivariable analysis for overall survival (OS) by adjuvant chemotherapy stratified by province. Abbreviations: ACT, adjuvant chemotherapy; PS, performance scale; CEA, carci-noembryonic antigen; CRM, circumferential resection margin.

Adjuvant chemotherapy was associated with improved OS hazard ratios for most categories of age, ECOG PS, pathologic stage, and CRM with significant improvements for those with age < 65 or ≥65 years, ECOG PS 0/I, pathologic stage 0, and those with a CRM > 1 mm or CRM ≤ 1 mm (Table 4 and Figure 5).

Oxaliplatin-based chemotherapy was not associated with improved OS in univariate or multivariable analyses.

## 4. Discussion

The standard regimen for patients with locally advanced rectal cancer (LARC) has traditionally been preoperative short-course radiotherapy (SCRT) or long-course radiotherapy administered in combination with fluoropyrimidine-based chemotherapy (CRT), total mesorectal excision, and postoperative AC [32,33,34,35,36,37]. There has been recent interest in identifying optimal preoperative radiotherapy regimens [11,15] but there is a relative paucity in further examination of AC due to the failure of most AC trials to demonstrate a significant benefit for DFS and OS [22,23,24]. The utility of AC has been demonstrated in noteworthy examples, including QUASAR, which showed benefits to OS and recurrence following a levamisole/fluorouracil-based regimen, and ADORE, which showed improved DFS using a FOLFOX protocol [27,38]. Recently, the field of rectal cancer has shifted its focus to TNT as an alternative to nCRT and includes the delivery of multiagent chemotherapy and neoadjuvant (chemo)radiotherapy prior to surgical resection or non-operative management [14,28]. Importantly, a number of these studies incorporate AC within their respective study designs in addition to TNT [13,14].

In this study, we performed a large, real-world, retrospective analysis from multiple Canadian institutions (academic and community) investigating the role of AC and its impact on clinical outcomes patients with LARC who received neoadjuvant CRT and rectal surgery. The usage of AC in our population was higher than expected with approximately 75% receiving postoperative chemotherapy (40% received oxaliplatin-based AC) compared to other studies suggesting rates of ~50% [27,39,40,41]. This usage of oxaliplatin-based regimens in our cohort may have contributed to the improvement in DFS and OS (compared with no AC) as supported by the results from the ADORE trial [27]. As expected, younger and fitter patients were more likely to receive AC as were those who achieved less downstaging to nCRT (more resistant disease) [42,43]. While elderly patients (>65 years) had worse OS in our cohort, there was no difference in DFS. This is likely explained by the fact that younger patients live longer, but AC did not improve local and distant recurrence differentially by age. The reasons behind the difference in likelihood of receipt of AC in older patients are likely multifactorial and beyond the scope of the current study but an interesting area for future investigation. Reassuringly, our Canadian population showed very similar local recurrence rates, 5-year DFS, and 5-year OS to those reported in the original German rectal trial [9].

As compared with large administrative databases, our study collected important prognostic variables and confirmed the important implications of performance status, pathologic stage, pretreatment CEA levels, and CRM involvement. When controlling for these factors, AC was significantly associated with DFS and OS with 40% and 54% improvements in survival, respectively. Further, we attempted to identify subgroups that may benefit more from adjuvant chemotherapy than others. Clear predictive subgroups could not be identified. There were, however, improvements in hazard ratios with the use of AC for most poor prognostic groups which suggests that there may be some modification of risk for these higher-risk settings.

There is a growing interest in identifying tissue, molecular, and radiographic markers in relation to response to treatment and prognosis in rectal cancer [44,45,46,47,48,49,50,51,52]. The majority of work to date has focused on identifying markers in the neoadjuvant setting while there is a relative sparsity of tumor-specific markers to guide clinical decision making regarding adjuvant chemotherapy. One emerging biomarker of interest is levels of circulating tumor DNA (ctDNA) [53,54]. There have been recent studies examining the role of this biomarker and liquid biopsies in CRC, namely its utility in prognosis as well as guiding treatment decision making [55]. The role of ctDNA in rectal cancer is also of growing interest [56]. One prospective study examining ctDNA levels in LARC before and after surgery detected ctDNA in 12% of patients 4–10 weeks following TME [57]. Detection of postsurgical ctDNA was associated with significantly lower recurrence-free survival and higher recurrence risk with or without adjuvant chemotherapy (HR 10 vs. 22, respectively) [57]. Subsequent work in LARC has demonstrated comparable findings [56,58,59,60]. The prospects for new biomarkers, including ctDNA, obtainable by minimally invasive methods such as liquid biopsies, show promise in identifying patients who may benefit most from adjuvant chemotherapy. Currently however, there are no definitive recommendations to incorporate ctDNA in routine clinical assessment in patients with LARC. Randomized control trials examining the predictive role of ctDNA in the context of adjuvant chemotherapy for LARC are required.

Our study is the largest reported Canadian experience examining the role of adjuvant chemotherapy in LARC. In addition to the large sample size, the granularity of our data, the median follow-up time, and the multi-institutional nature enhance the validity of our results. In the context of our study design, we recognize that the non-randomized nature of our data is unable to control for unknown and unmeasured confounding variables. Similarly, there was not an equal distribution of patients receiving and not receiving AC. Additionally, given the retrospective nature of this study, chemotherapy dose intensity/cycle numbers were not obtained and were beyond the scope of this study. Furthermore, this study includes patients who have received variable AC regimens left to the discretion of the treating physician. While this may reflect real-world practice during the timeframe of this study, there may have been unknown selection biases with respect to use of AC and type of AC.

To put our results in context in the contemporary treatment of LARC, nCRT followed TME and consideration of AC remains an acceptable treatment strategy and is considered in treatment discussions in Canada and remains part of recent guidelines around the world [28,29,61,62,63]. To date, only the PRODIGE 23 and STELLAR clinical trials have improved OS compared with this strategy and it is used infrequently at present [13,15]. Further, some patients initially thought to have early T2 or low-risk T3 disease (and treated with surgical resection up front or neoadjuvant short course radiation +/− delayed surgery) may have pathologic lymph node involvement requiring decisions regarding the role of AC. Similarly, some patients may wish to avoid upfront multiagent chemotherapy unless it is deemed necessary based on pathologic findings. Moreover, access to TNT strategies, which require timely coordination of multiple disciplines, may not be possible in resource-limited settings and therefore nCRT and AC may be preferred. Consequently, our real-world data adds important support for the use of AC after nCRT.

## 5. Conclusions

Although the management of LARC continues to evolve, questions regarding the use of AC remain relevant. Our large real-world data adds support to the use of AC if nCRT is utilized prior to surgical resection. While we demonstrate improvement to OS and DFS in these patients, our analysis could not identify clear subgroups that benefit from adjuvant chemotherapy.

## Figures and Tables

**Table 1:** Patient demographic and clinical characteristics by adjuvant chemotherapy.

			Adjuvant CT		
Characteristic		Total (N = 1448)	No (N = 363, 25.1%)	Yes (N = 1085, 74.9%)	*p*-Value
Province, n (%)	AB	606, 42%	150, 41%	456, 42%	<0.001
	BC	252, 17%	80, 22%	172, 16%	
	NL	188, 13%	22, 6%	166, 16%	
	ON	402, 28%	111, 31%	291, 27%	
Age, years	median (range)	61 (22–92)	66 (27–92)	60 (22–86)	
	≥65, n (%)	560, 39%	188, 52%	372, 34%	<0.001
Female, n (%)		438, 30%	111, 31%	327, 30%	0.874
BMI, kg/m^2^	Underweight	28, 1.93%	5, 1.38%	23, 2.12%	0.001
	Normal weight	432, 29.83%	90, 24.79%	243, 31.52%	
	Overweight	519, 35.84%	124, 34.16%	395, 36.41%	
	Obese	366, 25.28%	104, 26.65%	262, 24.15%	
	Unknown	103, 7.11%	40, 11.02%	63, 5.81%	
ECOG PS, n (%)	0	673, 46%	150, 41%	523, 48%	0.013
	1	524, 36%	133, 37%	391, 36%	
	2+	66, 5%	25, 7%	41, 4%	
	Unknown	185, 13%	55, 15%	130, 12%	
Distance from anal verge, cm	<5	478, 33%	123, 34%	355, 33%	0.927
	5–10	595, 41%	149, 41%	446, 41%	
	>10	273, 19%	68, 19%	205, 19%	
	Unknown	102, 7%	23, 6%	79, 7%	
Pretreatment CEA, ng/mL	<5	778, 54%	194, 54%	584, 54%	0.107
	≥5	497, 35%	115, 32%	382, 35%	
	Unknown	173, 12%	54, 15%	119, 11%	
Clinical stage, n (%)	II	439, 30%	108, 30%	331, 31%	0.787
	III	1009, 70%	255, 70.2%	754, 69%	
Radiation therapy dose, n (%)	<44	43, 3%	23, 6%	20, 2%	<0.001
	≥44 to 46	248, 17%	71, 20%	177, 16%	
	≥46	1157, 80%	269, 74%	888, 82%	
Surgery type, n (%)	LAR	737, 51%	185, 51%	552, 51%	0.964
	APR	665, 46%	165, 45%	500, 46%	
	PE	35, 2%	10, 3%	25, 2%	
	Unknown	11, 1%	2, 1%	8, 1%	
Quirke grade, n (%)	Good	677, 47%	168, 46%	509, 47%	0.913
	Moderate	177, 12%	43, 12%	134, 12%	
	Poor	91, 6%	21, 6%	70, 6%	
	Not recorded	503, 35%	131, 36%	372, 35%	
Pathological grade, n (%)	0	80, 22%	172, 16%	252, 17%	0.060
	I	67, 18%	206, 19%	273, 19%	
	II	110, 30%	327, 30%	437, 30%	
	III	106, 29%	379, 34%	485, 33%	
	IV	0, 0%	1, 0%	1, 0%	
Downstaged, n (%)	No	640, 44%	141, 39%	499, 46%	0.010
	Yes, not pCR	556, 38%	142, 39%	414, 38%	
	pCR	252, 17%	80, 22%	172, 16%	
CRM, n (%)	>1 mm; CRM not involved	1217, 84%	306, 84%	911, 84%	0.153
	≤1 mm; CRM involved	112, 8%	34, 9%	78, 7%	
	Not available	119, 8%	23, 6%	96, 9%	
Local pelvic recurrence, n (%)	No	1332, 92%	332, 91%	1000, 92%	0.216
	Yes	115, 8%	30, 8%	85, 7%	
Distant recurrence, n (%)	No	1132, 78%	289, 80%	843, 78%	0.444
	Yes	316, 21%	74, 20%	242, 22%	
Dead, n (%)	No	1130, 78%	244, 67%	886, 82%	<0.001
	Yes	318, 22%	119, 32%	199, 18%	

Abbreviations: AB, Alberta; BC, British Colombia; NL, Newfoundland and Labrador; ON, Ontario; ECOG PS, Eastern Cooperative Oncology Group performance status; CT, chemotherapy; CEA, carcinoembryonic antigen; BMI, body mass index; pCR, pathological complete response; CRM, circumferential resection margin; APR, abdominoperineal resection; LAR, low anterior resection; PE, pelvic exenteration.

**Table 2:** Univariate analysis of OS and DFS.

		OS			DFS		
		HR	(95% CI)	*p*-Value	HR	(95% CI)	*p*-Value
Adjuvant CT	No	1.00			1.00		
	Yes	0.50	0.4–0.63	<0.001	0.71	0.58–0.86	0.001
Province	AB	1.00			1.00		
	BC	1.26	0.96–1.65	0.10	1.41	1.11–1.78	0.004
	NL	1.34	0.88–2.02	0.17	1.58	1.15–2.16	0.004
	ON	0.47	0.35–0.64	<0.001	0.65	0.51–0.83	<0.001
Age at diagnosis	<65	1.00			1.00		
	≥65	1.67	1.34–2.08	<0.001	1.30	1.08–1.56	0.005
ECOG PS	0	1.00			1.00		
	1	1.80	1.38–2.34	<0.001	1.44	1.17–1.78	0.001
	2+	3.27	2.13–5.02	<0.001	1.86	1.26–2.77	0.002
	Unknown	1.91	1.39–2.63	<0.001	1.56	1.2–2.04	0.001
Distance from anal verge	<5	1.00			1.00		
	5–10	0.89	0.69–1.14	0.35	0.92	0.74–1.14	0.434
	>10	0.66	0.47–0.93	0.02	0.77	0.59–1.01	0.063
	Unknown	1.51	1.02–2.23	0.04	1.61	1.16–2.22	0.004
Pretreatment CEA	<5	1.00			1.00		
(Ug/L)	≥5	1.88	1.49–2.39	<0.001	1.73	1.43–2.11	<0.001
	Unknown	1.38	0.97–1.96	0.07	1.42	1.07–1.89	0.015
RT dose	<44 Gy	1.00			1.00		
	44–46 Gy	1.14	0.60–2.14	0.69	1.09	0.63–1.87	0.764
	≥46 Gy	0.63	0.34–1.15	0.13	0.77	0.46–1.29	0.321
Type of surgery	LAR	1.00			1.00		
	APR	1.36	1.08–1.71	0.01	1.30	1.08–1.57	0.006
	PE	2.43	1.43–4.16	<0.001	1.72	1.05–2.82	0.032
	Not reported	2.25	0.83–6.08	0.11	1.41	0.52–3.79	0.496
Quirke grade	Good	1.00			1.00		
	Poor	1.44	1.01–2.06	0.04	1.25	0.93–1.68	0.142
	Moderate	1.97	1.30–2.97	<0.001	1.68	1.18–2.4	0.004
	Not reported	1.36	1.06–1.74	0.020	1.25	1.02–1.54	0.033
Pathological stage	0	1.00			1.00		
	1	1.23	0.68–2.22	0.50	1.23	0.76–2.01	0.4
	2	3.27	2.00–5.33	<0.001	3.32	2.21–4.98	<0.001
	3	5.43	3.38–8.72	<0.001	6.53	4.42–9.65	<0.001
	4	31.16	4.15–233.89	<0.001	164.93	21.8–1248.04	<0.001
CRM involved	No	1.00			1.00		
	Yes	3.18	2.37–4.25	<0.001	3.10	2.41–4	<0.001
	Not reported	1.03	0.67–1.59	0.90	0.86	0.5823323–1.26	0.426
pCR	No	1.00			1.00		
	Yes	0.28	0.18–0.45	<0.001	0.26	0.18–0.38	<0.001
Downstaged	No	1.00			1.00		
	Yes	0.38	0.30–0.48	<0.001	0.32	0.26–0.39	<0.001

Abbreviations: OS, overall survival; DFS, disease-free survival; ECOG PS, Eastern Cooperative Oncology Group performance status; CT, chemotherapy; CEA, carcinoembryonic antigen; RT, radiotherapy; BMI, body mass index; pCR, pathological complete response; CRM, circumferential resection margin; APR, abdominoperineal resection; LAR, low anterior resection; PE, pelvic exenteration.

**Table 3:** Multivariate analysis for DFS by adjuvant chemotherapy.

				Adjuvant CT			
		No			Yes		
DFS		HR	(95% CI)	*p*-Value	HR	(95% CI)	*p*-Value
Pretreatment CEA	<5	1.00			1.00		
(µg/L)	≥5	1.67	1.15–2.43	0.007	1.47	1.16–1.86	0.002
	Unknown	1.58	0.95–2.64	0.079	1.59	1.11–2.29	0.012
Pathological stage	0	1.00			1.00		
	1	1.02	0.46–2.27	0.954	1.40	0.73–2.65	0.308
	2	2.04	1.07–3.89	0.029	3.44	1.98–5.98	0.00
	3	5.77	3.11–10.70	0.00	6.42	3.73–11.05	0.00
CRM	No	1.00					
	Yes	1.85	1.18–2.89	0.007	2.05	1.48–2.83	0.00
	Unknown	0.78	0.38–1.64	0.517	0.88	0.53–1.46	0.617

Abbreviations: DFS, disease-free survival; CT, chemotherapy; ECOG PS, Eastern Cooperative Oncology Group performance status; CEA, carcinoembryonic antigen; CRM, circumferential resection margin.

**Table 4:** Multivariate analysis for OS by adjuvant chemotherapy.

					Adjuvant CT		
		No			Yes		
OS		HR	(95% CI)	*p*-Value	HR	(95% CI)	*p*-Value
Age at diagnosis	<65 yo	1.00			1.00		
	≥65 yo	1.57	1.06–2.33	0.023	1.37	1.03–1.82	0.033
ECOG PS	0	1.00			1.00		
	1	1.15	0.72–1.83	0.551	1.52	1.07–2.16	0.02
	2+	1.93	0.98–3.78	0.056	2.51	1.36–4.64	0.003
	Unknown	1.21	0.66–2.20	0.535	1.19	0.75–1.89	0.464
Pretreatment CEA	<5	1.00			1.00		
(ug/L)	≥5	1.62	1.08–2.43	0.019	1.62	1.20–2.20	0.002
	Unknown	1.36	0.75–2.46	0.316	1.65	1.04–2.64	0.035
Pathological stage	0	1.00			1.00		
	1	0.95	0.41–2.22	0.915	1.76	0.71–4.34	0.22
	2	1.51	0.75–3.05	0.247	4.57	2.07–10.09	<0.001
	3	3.73	1.93–7.23	<0.001	6.74	3.07–14.78	<0.001
CRM	No	1.00			1.00		
	Yes	2.21	1.35–3.62	0.002	1.82	1.21–2.73	0.004
	Unknown	0.85	0.38–1.89	0.687	1.16	0.65–2.08	0.62

Abbreviations: OS, overall survival; CT, chemotherapy; ECOG PS, Eastern Cooperative Oncology Group performance status; CEA, carcinoembryonic antigen; CRM, circumferential resection margin.

## Data Availability

The data that support the findings of this study are available on request from the corresponding author, M.M.V. The data are not publicly available due to patient privacy/ethical concerns.

## Appendix A

**Table A1:** Hazard ratio and confidence interval for DFS.

Characteristic	Hazard Ratio (HR)	Confidence Interval (95% CI)
ACT (yes)	0.60	0.49–0.74
Age > 65 years	1.14	0.94−1.37
PS 1	1.17	0.94–1.46
PS 2+	1.27	0.85–1.92
PS not reported	1.09	0.80–1.48
CEA ≥ 5 µg/L	1.52	1.25–1.86
CEA not available	1.55	1.15–2.07
Pathology Stage 1	1.16	0.71–1.89
Pathology Stage 2	2.68	1.78–4.04
Pathology Stage 3	5.65	3.80–8.41
CRM < 1 mm/not involved	2.01	1.55–2.62
CRM not available	0.86	0.57–1.29

Abbreviations: DFS, disease-free survival; ACT, adjuvant chemotherapy; PS, performance status; CEA, carcinoembryonic antigen; CRM, circumferential resection margin.

**Table A2:** Hazard ratio and confidence interval for OS.

Characteristic	Hazard Ratio (HR)	Confidence Interval (95% CI)
ACT (yes)	0.46	0.36–0.58
Age > 65 years	1.45	1.16–1.82
PS 1	1.46	1.10–1.93
PS 2+	2.17	1.38–3.39
PS not reported	1.22	0.85–1.75
CEA ≥ 5 µg/L	1.63	1.28–2.08
CEA not available	1.55	1.08–2.22
Pathology Stage 1	1.23	0.67–2.23
Pathology Stage 2	2.66	1.61–4.41
Pathology Stage 3	4.74	2.90–7.76
Pathology Stage 4	71.08	9.22–547.72
CRM < 1 mm/not involved	2.11	1.56–2.86
CRM not available	1.04	0.65–1.65

Abbreviations: OS, overall survival; ACT, adjuvant chemotherapy; PS, performance status; CEA, carcinoembryonic antigen; CRM, circumferential resection margin.
